# Comprehensive psychosocial and spiritual care of people with advanced chronic conditions: The experience of La Caixa foundation program at 15 years

**DOI:** 10.1017/S1478951525101405

**Published:** 2026-01-28

**Authors:** Xavier Gomez-Batiste, Rosa Montoliu, Montserrat Codinachs, Cristian Tebé, Jorge Maté-Mendez

**Affiliations:** 1 Scientific Director, Program for the Comprehensive care of people with advanced chronic conditions, La Caixa Foundation, Barcelona, Spain; 2 Professor and Chair of Palliative Care, Faculty of Medicine, University of Vic/Central Catalonia, Vic, Barcelona, Spain; 3 Clinical Information and Documentation Department, Institut Català d’Oncologia, Barcelona, Spain; 4 Deputy Director Chair of Palliative Care, Faculty of Medicine, Universitat de Vic/Central Catalonia, Catalonia, Spain; 5 Biostadistics Support and research unit, Germans Trias i Pujol Research Institute and Hospital (IGTP), Badalona, Spain; 6 Clinical Care Coordinator, Psycho-Oncology Service, Institut Català d’Oncologia, Barcelona, Spain

**Keywords:** Psychosocial and spiritual care, advanced chronic patients, care model, psychosocial interventions, real-world

## Abstract

**Objectives:**

To describe and assess the overall results of the La Caixa Foundation and the ICO/UVIC Chair of Palliative Care (Former WHO Collaborating Centre) Program “Comprehensive Care of People with Advanced Chronic Conditions” at 15 years (2008–2023).

**Methods:**

We used qualitative and quantitative methods, such as prospective, quasi-experimental, and pre-post test designs, to evaluate the effectiveness of the interventions led by psychosocial teams providing support to existing healthcare services. Data were collected from the Program’s unique shared online information system, retrieving output and outcomes information, including data obtained from validated psychosocial evaluation instruments and semi-structured interviews with patients, relatives, professionals and other stakeholders, focusing on effectiveness, satisfaction, and perceived quality of different aspects of the Program, as well as outputs.

**Results:**

From 2008 to 2022, the Program implemented 65 teams in Spain and 11 in Portugal across all the provinces, with 379 full-time professionals. They saw 286,644 patients and 371,023 relatives, with a median intervention duration of 2.3 weeks. Patients’ mean (SD) age was 73.2 (14.9) years; 52.3% were women, and most had a cancer diagnosis (60.1%). After 3 consecutive interventions, patients showed significantly improved psychosocial parameters, according to the Assessment of PSS Needs (ENP-E) and Existential Loneliness Detection Scale (EDSOL). Patients, relatives, and stakeholders were highly satisfied. The Program has developed a Master’s degree that has trained over 250 professionals and conducted 371 courses/workshops and 302 lectures. The Program developed tools, manuals, and protocols that were published, available, and common to all professionals involved. It also developed innovative approaches responding to special settings and needs.

**Significance of results:**

A care program within a collaborative framework between public health services and non-profit foundations is an effective, efficient, and feasible model for organizing the psychosocial and spiritual dimension of care for patients with advanced chronic conditions and their relatives.

## Introduction

The psychosocial and spiritual (PSS) needs of people with advanced chronic conditions (PACC) and those at the end of life (EoL) are considered essential needs and relevant components of care (Holland et al. 1998; Ferris et al. 2007; Gil Moncayo et al. 2013; Maté-Méndez et al. 2013; Gómez-Batiste et al. 2017; Fraguell et al. 2018). Although most palliative care models include these dimensions, many experiences were initially focused on symptom control (i.e., pain), and the roles of specific professionals addressing the psychosocial dimensions, such as psychologists, social workers, chaplains, and others, developed gradually. Consequently, PSS needs were partially met in the initial phases of palliative care implementation. Additionally, in the initial history of palliative care implementation in many countries, knowledge was limited, and validated assessment instruments were lacking. Other program components, including volunteers and complementary therapies, such as art, music, or occupational therapy, faced the same challenges and were developed gradually.

In Spain, palliative care development has experienced the same limitations, with psychologists, social workers, or chaplains frequently absent during the initial implementation. Although pioneers and leaders in the field intensively trained and mentored specialized professionals, their implementation within the services was still gradual (Bayés et al. 1996, 1997). Despite these limitations, the Catalonia Cancer Plan for 1992–1994 supported the creation of 24 Psycho-Oncology Units for non-advanced and advanced cancer patients as well as their relatives. Thanks to this institutional support, the first Psycho-Oncology Unit in Catalonia was established at the end of 1998 at Duran i Reynals Hospital (Catalan Institute of Oncology) (Gil 2000). Three years later, the 2001–2004 Catalonia Cancer Master Plan included among its objectives the provision of psycho-oncology professionals to Catalonia’s leading cancer centers, recognizing psychological care as an essential component of oncological care that must be integrated into the healthcare system. Currently, this first unit has evolved into the Psycho-Oncology Service, directed by Francisco Luis Gil Moncayo, and collaborates with the Spanish Ministry of Health in defining recommendations for psychological care (‘Cataluña guía la expansión de la psicooncología pública en España’ 2024). Despite these advances, the implementation of psycho-oncological support in our country has been uneven.

Many care strategies, policies, and tools have been implemented in the last decades to address PSS needs (Callahan 2000; Breitbart and Heller 2003; Chochinov 2007; Puchalski et al. 2009, 2014). In 2008, a qualitative assessment in Spain identified some weaknesses, leading to the development of areas addressing these needs (Gómez-Batiste et al. 2010).

## La Caixa foundation

La Caixa Foundation (LCF) has 125 years of experience, with the main mission of improving care for society and, especially, for vulnerable people (La Caixa Foundation, n.d.-b). LCF plays a catalytic role by identifying unmet needs and implementing programs to generate experience and evidence that can be extended across the system and society. The LCF has developed several programs for vulnerable people, such as the elderly, children at risk of social exclusion, populations with long-standing unemployment, and situations of unwanted and existential loneliness and poverty, considering gender inequalities (La Caixa Foundation, n.d.-c). LCF’s global budget is more than 600 million euros/year, 320 million euros/year devoted to social programs, and it is the 3rd social foundation in the world focused on improving society.

LCF identified areas of improvement of the palliative care model in Spain and designed a *“Program for the Comprehensive Care of People with Advanced Chronic Conditions”* (Program) based on its values and mission (La Caixa Foundation, n.d.-a). The Program started in 2008 and focused on PSS needs, with the support of the World Health Organization (WHO), and was included in the Spanish national strategy for palliative care of the Spanish Ministry of Health (Ministerio de Sanidad, n.d.).

## The ICO/UVIC chair of palliative care

The ICO/UVIC Chair of Palliative Care (CPC) was the first CPC in Spain, created from the Qualy Observatory as a WHO Collaborating Centre at the *Institut Català d’Oncologia* (ICO, Catalan Institute of Oncology) in 2012, to develop the academic aspects of palliative care (Gómez-Batiste et al. 2019). It has developed undergraduate and postgraduate training, research, and consultation focused on chronic palliative care. The CPC is responsible for the Program’s Scientific Direction, which encompasses design, support to implementation and evaluation, quality improvement, and training.

## Program overview

### Origins, mission, and vision

The Program was created in 2008, owing to existing evidence that our Spanish social and health system did not cover the PSS needs of PACC and those at the EoL (>50% being complex patients), and PSS interventions were poorly implemented in palliative care services. Preliminary and interim results of the Program have been published elsewhere (Gómez-Batiste et al. 2011; Mateo-Ortega et al. 2013, 2018).

The Program’s mission is to alleviate the suffering of PACC by addressing their PSS needs as essential components of comprehensive care. The vision is to recognize PSS care as essential to palliative care, considering it a human right and ensuring universal access for PACC. Achieving this goal may require generating experience and evidence to strengthen advocacy efforts aimed at encouraging policymakers to insert this approach into health and social systems.

### Values and principles

The Program shares the FLC values of compassion, humanitarianism, and care for vulnerable persons at EoL and is guided by the principles of a community-oriented approach and recognition of universal accessibility and high-quality PSS care as fundamental human rights.

### Objectives

The Program’s objective is to develop experience and evidence about the effectiveness of PSS needs assessments and care interventions in patients at EoL. The Program aims to promote the recognition of comprehensive palliative care – including its PSS components – as a human right for people at EoL by generating and disseminating care experiences, evidence, and advocacy efforts to promote the integration of this policy into the health care system.

## Models of care and organization

### Model of care and intervention

The model of care is defined as the comprehensive assessment and care of complex PSS needs of EoL patients and their families, integrated in palliative generalist or specialist care provision and supporting professionals looking after them.

### Model of organization

The organization model consists of multidisciplinary psychosocial teams, comprised of 2–3 psychologists and 1–2 social workers, called EAPS. (*Equipo de atención psicosocial* in Spanish). These teams are collaborating with existing palliative care or other generalist services (i.e., hospitals, nursing homes, primary care services), with a formal agreement to complement their care and, thus, support them. This model is implemented in a specific territory.

### EAPS providers and recipient services

The providers of the EAPS are non-profit organizations, and the recipient services are hospitals, palliative care services, primary care services, and/or nursing homes in the district. The providers and the recipient institution sign a formal agreement. Most EAPS care for different types of patients, with 3 additional specific EAPS for pediatric patients, another one for patients with lateral sclerosis, and another one for patients with cancer.

### LCF and CPC managerial models

The Direction and a multidisciplinary team of 10 full-time members at the LCF provide support, advice, and follow-up to the EAPS, with frequent interactions. Additionally, a team from the Chair of Palliative Care UVIC/UCC, which consists of experts in different areas, acts as Scientific Director (in Spain). Additionally, a recognized and solvent audit firm performs an external independent auditing process annually to oversee organizational and economic aspects and to assess stakeholders’ perceptions of psychosocial teams (with a score of 9 out of 10 in 2022).

### Definition, identification, and evaluation of target patients

The target patients are defined by the combination of an advanced chronic condition and a limited life prognosis, with especially complex PSS needs or vulnerability. These needs are identified using validated screening tools and assessed systematically during follow-up using validated tools and formal protocols (see below). The screening tools include the Detection of Emotional Distress (DME, *Detección de Malestar Emocional*), the DME-caregivers (DME-C), the Assessment of PSS Needs (ENP-E, *Evaluación de Necesidades Psicosociales y Espirituales*), and the Spiritual Exploration and Assessment Guide questionnaire (GES, *Guía de Exploración y Evaluación Espiritual*) (Limonero et al. 2012, Galiana et al. 2014; Mateo-Ortega et al. 2019; Limonero et al. 2023).

LCF fully funds the EAPS, which are therefore completely free of charge for patients and families.

### Recognitions

The Program is included in the Spanish Palliative Care National Plan with all the regional Health Care Departments, under a formal agreement. It was initially included as an activity of the WHO Collaborating Centre for Public Health Palliative Care Programs at the Catalan Institute of Oncology (WHOCC-ICO) (2008–2020) and of the CPC after 2012. The European Council has also recognized it and, more recently, it was granted the 2024 International Association for Hospice and Palliative Care (IAHPC) Institutional Award.

### Recruitment and training of professionals

Professionals in the EAPS are recruited and trained following a systematic approach, starting with the selection of providers in the NPO sector. The selection of leaders and the recruitment, training, coaching, benchmarking, and follow-up of teams were included to enhance clinical competence, promote a team-based approach, and reinforce values and behaviours of commitment and compassion. This strategy also aimed to strengthen the teams’ capacity to implement a complex Program encompassing multiple institutions and services, following a quality improvement approach and a population-based, community-oriented perspective.

### Psychosocial complexity

To effectively address these PSS needs, it is crucial to understand and operationalize the concept of psychosocial complexity. In the context of cancer and advanced chronic conditions, this multidimensional construct refers to situations in which patients experience an increased sense of threat, leading to difficulties in coping with their illness, particularly when the prognosis is poor or uncertain. Such emotional distress may result in poorly adjusted traumatic reactions, which can be exacerbated by adverse social determinants such as limited family support or social exclusion, thereby increasing the overall complexity of the case (Casellas-Grau et al. 2021).

The study by Casellas-Grau and colleagues (Casellas-Grau et al. 2021), using the Delphi method, reached a consensus on the definition of this complexity and identified its 4 main factors: medical-physical, social-family, psychological, and spiritual. It also proposed a list of indicators for its identification. The Program deployed the intervention within this conceptual framework. By implementing multidisciplinary teams of psychologists and social workers (EAPS) that systematically assess and address these complex needs using validated instruments, the Program represents a practical, large-scale application of the consensual principles for managing psychosocial complexity.

This study aimed to describe the implementation of the LCF Program for the Comprehensive Care of PACC and to summarize its main results at 15 years (2008–2023). This article not only describes the Program’s implementation and outcomes but also provides real-world evidence on the effectiveness of a care model specifically designed to address psychosocial complexity in patients with advanced chronic conditions and their families.

## Methods

### Model of evaluation

Given the Program’s aim of generating experience and evidence, it has developed a comprehensive and systematic evaluation process, combining quantitative and qualitative methodologies. The objective is to assess the Program’s effectiveness, perceived quality, and usefulness among patients, relatives, professionals receiving support, stakeholders, and participants in the School of carers (informal caregivers). The evaluation procedures established and performed to date are summarized in Table 1.

**Table 1.** Evaluation of the Program performed in the context of the comprehensive and systematic evaluation process

Study	Design/variables and outcomes	Population	Year	Relevant results
**Quantitative methodology**				
Activity and characteristics	Activity (outputs)	Patients	2010−2023	>300,000
	Population characteristics			Results (latest year):
	Intervention duration			Age (years), mean (SD): 73.8; 51.7% women.
	Place of the intervention			Pathology: 63% oncologic
				Intervention duration: 15 d.
				Place: 55% hospital
		Relatives	2010−2023	>480,000
				Results (latest year):
				Age (years), mean (SD): 56.8; 68.8% women.
				Pathology: 71.4% oncologic.
				Main caregiver: 74.7% women.
				Relationship with patient: 47.0% child
Effectiveness in psychosocial parameters – categorical or numeric scales	Observational, prospective, longitudinal, multicenter, quasi-experimental pre-post study using categorical or numeric scales for:	Patients	2010, 2011, 2012, 2015, 2016, 2016	>43,000 All parameters improved significantly in the 3 consecutive interventions.
	- Mood, discomfort, anxiety, suffering, adjustment, communication, meaning, peace, and forgiveness (patients).			
	- Anxiety, discomfort, depression, and insomnia (relatives).	Relatives	2011, 2012, 2015, 2016	>32,000 All parameters improved significantly in the 3 consecutive interventions.

Effectiveness in psychosocial parameters – validated tools (patients)^a^	Observational, prospective, longitudinal, multicenter, quasi-experimental pre-post study using validated tools:	Patients	2017, 2018, 2019	⁓ 13,000 patients All parameters improved significantly in the 3 consecutive interventions (visits).
	ESAS, ENP, ENP-E (in 2019 ESAS, ENP-E, EDSOL, and Barthel).			
Effectiveness in anxiety and distress (relatives)	Observational, prospective, longitudinal, multicenter, quasi-experimental pre-post study	Relatives	2011−2016	>105,000 relatives. All parameters improved significantly after 3 interventions.
	Anxiety and distress			
Social risk	Observational, descriptive, cross-sectional, multicenter study using the TSO instrument to assess:	Patients	2022, 2023	>10,000 patients Need help for ADL: 82.5% Live alone: 27.1% Live in inadequate homes: 26.3% Poverty criteria: 16.8%
	Family structure, social interactions, resources (formal and informal), ADL, economic issues, and housing characteristics.			
Spirituality	Survey using semi-structured interviews	Patients	2022, 2023	>48,000 patients
				Spiritual beliefs: 65.4%
				Beliefs help in adjusting to disease: 61.6%
				Willing to share with others: 17.3%
Spirituality – pilot study	Cross-sectional study to assess the feasibility of using the ENP and GES instruments to explore spirituality needs.	Patients	2016	215 patients The GES was identified as more complex to answer
	Qualitative survey to identify factors facilitating their use.			Facilitators: advanced training, patients’ openness
Perceived quality of care	Semi-structured phone survey	Patients	2013	77 patients
				Excellent care: 68.0%
				Recommend to other patients: 89.6%
				Most patients expressed that the care provided fulfilled their information and care expectations.
				Most appreciated features: accessibility, kindness, capacity to share and express, and continuity.
	Semi-structured survey including 84 items assessing specific care interventions.	Relatives	2014	346/1027 relatives responded
				Global perception of high quality of care
School of carers	Pre-post assessment of self-competencies in several dimensions: care, emotional, stress, dealing with practical and physical needs, utility, feasibility in practical care.	Caregivers	2021, 2022	20 caregivers:
				86.5% females, 61.5% child, 60.6% high education
				Caregiver role: 32.9% > 5 years, 32% full time
				56.9% had at least one stressful situation in the last month
				Perceived self-competencies score: 6.53 before vs. 8.12 after (*p* < 0.001).
				86% perceived increased capacity of care
				Utility of the training score: 8.51 (over 10)
				Recommend to other caregivers score: 9.11 (over 10)
Perceived quality and satisfaction – professionals of recipient services	Semi-structured survey using numerical 1−10 scales to assess quality of care for patients, utility of the intervention, global satisfaction, coordination, communication	Professionals at recipient services	2017−2023	1244/2191 (56.8%) responded in 2023
				Mean scores > 9.2 points.
				Quality of care scores: 9.4
Caregiver overload	Cross-sectional assessment using the Zarit scale	Relatives (caregivers)	2023	>3000 relatives
				58.7% are overloaded
**Qualitative methodology**				
Patients’ experiences, perspectives, and personal impact	Qualitative study using narrative semi-structured interviews	Patients	2017	Patients expressed relevant benefits and high satisfaction. Areas that may be improved: increase intervention duration, resources, social approach, and the professionals’ spiritual approach.
				Relevant factors for peaceful relationships and love with family and friends, symptom control, autonomy, and humour.

a Results regarding changes in ENP and EDSOL scales throughout 3 consecutive interventions are described in detail in the results section of the manuscript.

ADL, activities of daily living; EDSOL, Existential Loneliness Detection Scale (*Escala de Detección de la Soledad Existencial*); ENP, Assessment of Psychosocial Needs (*Evaluación de Necesidades Psicosociales*); ENP-E; Assessment of Psychosocial and Spiritual Needs (*Evaluación de Necesidades Psicosociales y Espirituales*); ESAS, Edmonton Symptom Assessment System; GES; Spiritual Exploration and Assessment Guide questionnaire (*Guía de Exploración y Evaluación Espiritual*); SD, standard deviation; TSO, Social Needs Tool.

This manuscript focuses on the implementation results over 15 years and provides examples of some of the most relevant effectiveness outcomes regarding patients’ PSS needs. Moreover, this manuscript summarizes other crucial aspects of the Program’s experience and outputs, including training professionals on EAPS, developing additional and innovative approaches, and generating tools, manuals, protocols, and publications within the Program framework.

### Follow-up and data collection

The Program has a unique shared online information system that retrieves all the output and outcomes data according to ethical and legal regulations.

### Protocolization

The care processes are protocolized and published into 2 manuals available to all professionals.

### Evaluation of the program’s effectiveness

The Program’s effectiveness in improving the psychosocial parameters of patients and relatives was systematically evaluated and monitored using validated tools, including psychosocial evaluation instruments (Psicpal): ENP-E, DME, DME-C, GES, Existential Loneliness Detection Scale (EDSOL, *Escala de Detección de la Soledad Existencial*), and the palliative Needs – version 4.0 (NECPAL 4.0, NECesidades PALiativas) (Càtedra de Cures Pal·liatives, n.d..; Limonero et al. 2012; Gómez-Batiste et al. 2013; Galiana et al. 2014; Viel Sirito et al. 2018; Mateo-Ortega et al. 2019; Limonero et al. 2023). The systematic evaluation also includes semi-structured quantitative surveys, qualitative interviews, and other assessments of patients, relatives, and professionals, as well as systematic and continued assessments of perceived quality and added values of all receptor services and stakeholders (Table 1).

### Ethical considerations

The Program and its information system, evaluation processes and methodologies, and use of information have been formally approved by the Ethics Committee of the IDIBELL Institute and the UVIC/UCC.

### Statistical analysis

Cohort characteristics were summarized using counts and percentages for categorical variables. Continuous variables were reported as mean and standard deviation (SD) or as median and interquartile range (IQR), depending on their distribution.

To evaluate effectiveness, pre-post comparisons of ENP-E and EDSOL scores between the first and third visits were conducted using paired *t*-tests. Differences were reported with 95% confidence intervals (CIs). All analyses were performed using R software (version 4.3.0), with a two-sided significance level set at 0.05.

## Results

### Program implementation and coverage

Between 2008 and 2022 (15 years), the Program implemented 65 teams (EAPS) in Spain and 11 in Portugal, with a total of 379 full-time professionals. They provided care to 286,644 patients (38,560 in the last year), 371,023 relatives (44,115 in the last year), and provided support to 552 services (172 hospitals, 190 primary care provided at home, 190 nursing homes, and others). The median intervention duration was 2.3 weeks, and most patients (62%) died during the process of EAPS care.

**Figure 1.**
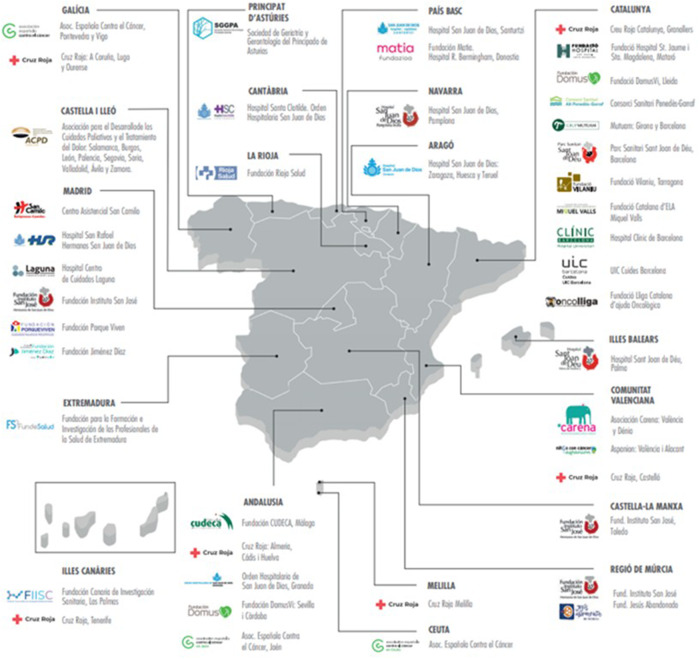
Distribution of the multidisciplinary psychosocial teams across Spain.

**Figure 2.**
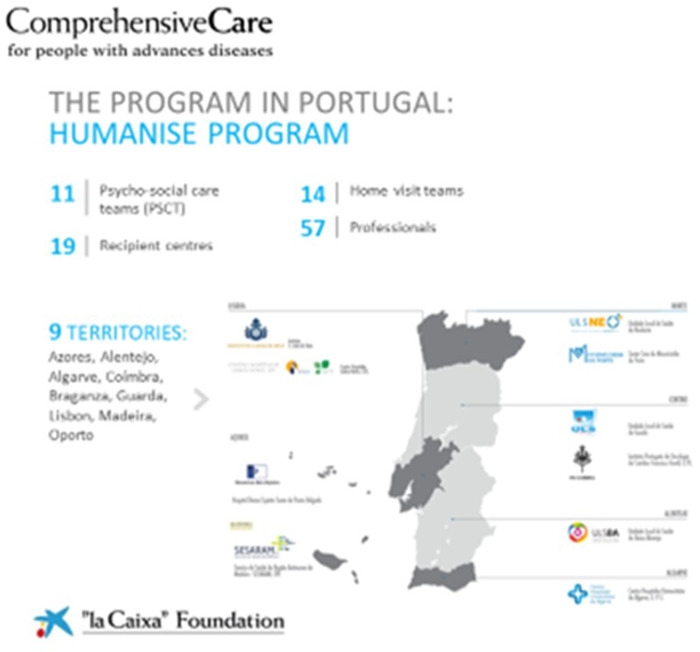
Distribution of the multidisciplinary psychosocial teams across Portugal.

The program is present in the 52 provinces in Spain (Fig. 1) and all 9 territories in Portugal (Fig. 2) and covers an estimated 30% of the total psychosocial needs in Spain.

### Patients’ characteristics

Among patients seen in 2023, mean (SD) age was 73.2 (14.9) years and 52.3% were women. The primary diagnosis was cancer (60.1%), followed by frailty (8.7%), dementia (3.9%), and organ failure (2%). Table 2 summarizes the demographic characteristics of patients according to cancer diagnosis.

**Table 2.** Demographic characteristics of patients overall and according to cancer diagnosis

	All *n* = 6471	Non-cancer *n* = 2531 (39.1%)	Cancer *n* = 3940 (60.9%)
**Gender, *n* (%)**			
Male	3084 (47.7)	1059 (41.8)	2025 (51.4)
Female	3387 (52.3)	1472 (58.2)	1.915 (48.6)
**Age (years)**			
*Mean, SD*	73.2 (14.9)	79.0 (14.7)	69.4 (13.8)
*Median (IQR)*	75.4 (63.5−84.8)	82.8 (72.4−89.4)	70.4 (60.5−79.8)

IQR, interquartile range; SD, standard deviation.

Over half of the patients (68.3%) had an advanced disease stage or were near EoL. Regarding their emotional state, 77.6% of patients reported experiencing a fair or poor mood, and 75.6% perceived their well-being as fair or poor. The PSS needs showed high or the highest complexity index in 68.1% of non-oncologic and 57.6% of oncologic patients, according to the ENP instrument. Regarding their social situation, 23% of the patients had limited support for care at home, and 30.1% had limited social interaction. Among non-oncologic patients, 16.1% reported a severe or high perception of existential loneliness.

### Effectiveness outcomes: Patient benefits

**Figure 3.**
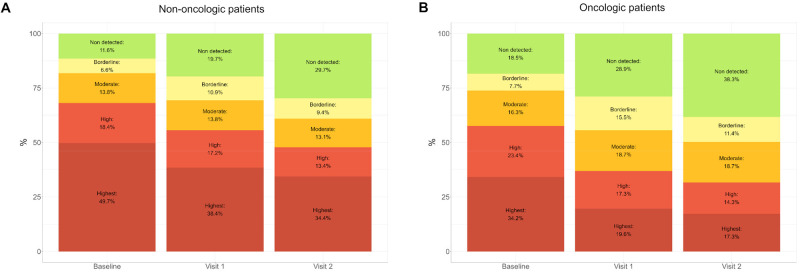
Evolution of the ENP-E tool complexity/severity levels in non-oncologic (A) and oncologic (B) patients throughout 3 consecutive interventions (visits). A quasi-experimental pre-post study was conducted to assess the Program’s effectiveness in improving palliative care needs, as described in Table 1. A total of 829 patients with 3 consecutive assessments were analyzed. The levels of complexity were constructed as described in (Mateo-Ortega et al. 2019). ENP-E, *Evaluación de Necesidades Psicosociales y Espirituales del Enfermo* (Evaluation of patient’s psychosocial and spiritual needs).

**Figure 4.**
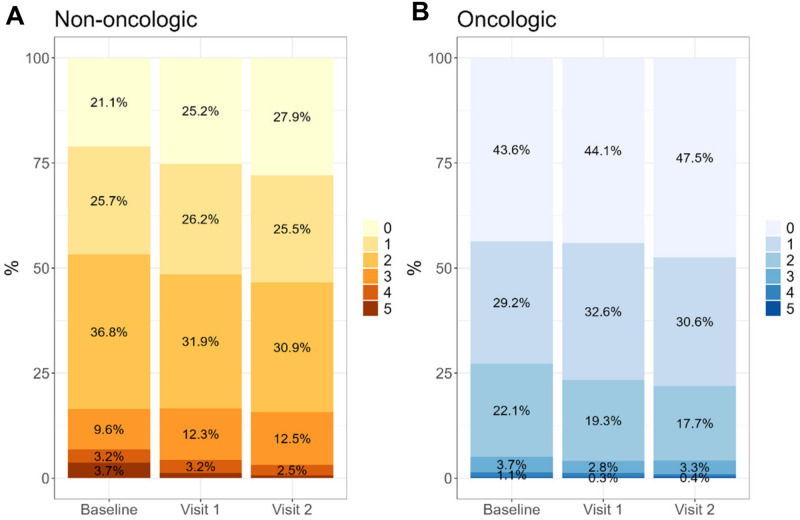
Evolution of the EDSOL scale scores measuring perceived existential loneliness, in non-oncologic (A) and oncologic (B) patients throughout 3 consecutive interventions (visits). A quasi-experimental pre-post study was conducted to assess the Program’s effectiveness in improving palliative care needs, as described in Table 1. A total of 1114 patients with 3 consecutive assessments were analyzed, with scores ranging from 0, no perception to 5, severe loneliness, as described in Sirito *et al*., 2018. EDSOL, *Escala de Detección de la Soledad Existencial* (Existencial Loneliness Detection Scale).

The Program’s effectiveness in improving patients’ PSS needs according to cancer diagnosis, including the ENP-E and the EDSOL parameters, is shown in Figs. 3 and 4. After 3 consecutive interventions (visits), patient classification shifted towards a lower level of complexity, with more patients in non-detected categories and a reduction in high and highest-level categories (Fig. 3). In oncology patients the mean baseline ENP-E total score was 33.34 (7.79), which decreased to 30.56 (7.29) at visit 1, resulting in a statistically significant mean difference of 2.8 points (95% CI: 2.3–3.3, *p* < 0.001). In non-oncology patients, the baseline mean score was 31.21 (6.96), which decreased to 29.03 (6.50) at visit 1, resulting in a statistically significant mean difference of 2.2 (95% CI: 1.9–2.5, *p* < 0.001). These results indicate that the program improved PSS needs in non-oncologic and oncologic patients. Likewise, the interventions resulted in a shift towards decreased EDSOL scores, indicating decreased existential loneliness in both patient groups, supporting the program’s effectiveness (Fig. 4).

### Effectiveness outcomes: Perceived quality by professionals and stakeholders

Professionals’ perceived quality was assessed using a semi-structured survey with scores ranging from 0 to 10, which was answered by 842 of 1663 professionals of recipient services. The mean score of 5 parameters assessed was 9.29 in 2022 (Table 1). In a survey of 261 stakeholders of the loneliness program described below, 79.1% strongly agreed that the program offers a high patient benefit.

### Training strategy for EAPS professionals

Training of EAPS professionals has been systematically and academically accredited as a *Master’s degree*, which is currently in its 9th edition (University of Vic/Central Catalonia (UVIC/UCC), n.d.). As a result of the program, more than 250 professionals (around 80% of the total) have been trained and have obtained their Master’s. A common clinical documentation and information system (available online) and the development and validation of tools, manuals, protocols, and recommendations (see curriculum) have enhanced the training and quality of care assurance.

### Training strategy for professionals

The training of professionals specialized in PSS areas has been one of the most relevant activities of the services in the Program, with 371 courses and workshops and 302 lectures. A total of 39,480 professionals were involved in these activities, which received a quality assessment score of 4 out of 5.

### Development of additional and innovative approaches

The Program has developed policies to respond to special needs, such as unwanted loneliness, among others, and settings (nursing homes), to adapt to events such as the COVID pandemic (Telematic care), and to educate and support caregivers.

### Unwanted and existential loneliness

A specific program focused on unwanted loneliness was created in 2015, aimed at identifying PACC and loneliness in the community and providing volunteer support. The organization of the loneliness program consists of appointing a coordinator who establishes agreements on care programs with the existing social organizations, from a community perspective. The program started in pilot areas in 2016 and is currently expanding across the Program system.

### Special patient’s needs: Paediatrics, motoneuron diseases, dementia, and COVID

To address the specific needs of especially complex ACP, some teams were developed to provide specific care to pediatric patients (3 EAPS teams) and patients with lateral sclerosis (1 EAPS). Additionally, all EAPS receive training to address the specific needs of these patients and those of people with dementia. During the COVID pandemic, teams were directed to focus on patients across all types of services, including intensive care units, emergency services, and nursing homes, and were trained to provide online care.

### Nursing homes

In 2021, PSS needs in nursing homes were identified as one of the most relevant unmet care areas. In response, a specific area of the Program was developed to address this setting. The model consisted of appointing and training additional EAPS team members to provide care in nursing homes, resulting in 65 new professionals recruited. The implementation of the Program, specifically in this setting, has contributed to advancing the current debate and establishing protocols for PSS interventions that may benefit people with advanced dementia, their relatives, and the professionals responsible for their care.

### Development of the community perspective

The EAPS teams are currently developing the community perspective, supported by the loneliness and volunteer programs, and their relationship with existing Compassionate Communities projects in Spain. Emerging challenges have been identified for successfully developing the community-based approach, particularly in identifying populations experiencing especially complex social vulnerability. This has led to the development of the concept of a “social vulnerability cluster,” defined by the combination of an advanced chronic condition, advanced age (>80), female gender, loneliness, poverty, limited access to services, and associated conditions, such as bereavement or abuse, especially in home settings or nursing homes. These populations frequently face limited access to both conventional and palliative care services, and are often and unnecessarily admitted to emergency services and hospital units at the EoL. To respond to the needs of patients in the social vulnerability cluster, the Program has strengthened its links with Compassionate Communities programs. Additionally, a community hospice project is currently being designed in Central Barcelona.

### Online care

The COVID pandemic raised the need to develop an online version of the Program to ensure the continuity of care. The online program was designed following a specific policy for online care and was implemented after specific training of EAPS professionals.

### School of carers and support

A “School of carers” was implemented within the Program to enhance and improve care by relatives, informal caregivers, and volunteers, promote their competence in addressing patients’ needs (whether emotional, practical or logistical), and identify the risk of complex bereavement and family claudication. In this regard, caregivers of PACC experience existential and emotional distress, leading to a significant caregiver burden. The School has developed a comprehensive online/presential education and training program (Càtedra de Cures Pal · liatives, n.d.). The School of carers was assessed as part of the systematic Program evaluation using a pre-post design (Table 1). Caregivers participating in the training program reported a significant increase in perceived self-competence and rated the program’s usefulness with a mean score of 8.51 out of 10.

### Tools, manuals, protocols, and publications

Several tools have been validated to assess needs and effectiveness (Barbero Gutiérrez et al., n.d..; Novellas Aguirre de Cárcer et al. 2018) and are systematically used (Maté et al. 2009; Viel Sirito et al. 2018; Limonero et al. 2019; Mateo-Ortega et al. 2019; Limonero et al. 2023). Two manuals have been edited to describe the model of intervention (Barbero Gutiérrez et al., n.d.; Novellas Aguirre de Cárcer et al. 2018).

## Discussion

A comprehensive program to respond to the PSS needs of patients with ACC and their families has been designed, funded, and implemented by the LCF for 15 years. To our knowledge, no other systematic, comprehensive, and large-scale program with such extended broad territorial coverage in Spain and Portugal has been developed to provide care to these patients.

Over the past 15 years, the Program has implemented numerous EAPS and supported different service types across all provinces in Spain, covering 30% of the country’s estimated needs. The Program has served over 800,000 patients and their relatives through more than 500 different services nationwide, reflecting extensive coverage and a critical mass of experience. A total of 76 dedicated psychosocial teams have been implemented (65 in Spain and 11 in Portugal), comprising 390 full-time professionals following a shared model of care and organization. The Program is systematically evaluated, and its organizational structure is based on centralized information and managerial systems. Its wide coverage, systematic evaluation, and organizational characteristics make this Program a unique initiative. The Program’s evaluation demonstrates its effectiveness in meeting the primary objective of improving the PSS dimensions of care. It is also perceived as improving the quality of life of patients and families and as providing valuable support to professionals and stakeholders.

Our findings on the Program’s effectiveness in addressing complex PSS needs resonate strongly with the recently established consensus framework on psychosocial complexity in cancer and advanced illness (Casellas-Grau et al. 2021). The significant improvements observed in ENP-E and EDSOL scores, alongside the reduction in complexity levels among our patients, provide robust empirical support for the conceptual model proposed by Casellas-Grau and colleagues. This model posits that psychosocial complexity is a multidimensional phenomenon arising from the interplay of medical-physical, psychological, social-family, and spiritual factors. The structure of our EAPS teams, comprising psychologists and social workers, directly mirrors this multifaceted approach, enabling comprehensive assessments and interventions that align with the consensus definition. Furthermore, the specific indicators of high complexity identified in the Delphi study, such as severe anxiety, despair, lack of a caregiver, and loss of meaning, are precisely the domains where our Program demonstrated the most impactful outcomes. Thus, the LCF Program can be viewed not only as a successful care model but also as a large-scale validation of the practical application of the psychosocial complexity construct, demonstrating that a targeted, multidisciplinary intervention can effectively mitigate its core components.

Several methodologies, including quantitative pre-post, qualitative, and mixed methods, have been applied to assess the effectiveness and the perceived quality of care among patients, relatives, professionals, and stakeholders sequentially in different phases of its evolution. The findings show improvement in patients’ quality of life, perceived well-being, and reduced suffering and loneliness.

Our study results are encouraging regarding the Program’s effectiveness, showing that after 3 consecutive interventions, patients demonstrated significant improvement in psychosocial parameters, according to the PSS Needs Assessment (ENP-E) and the Existential Loneliness Detection Scale (EDSOL). The success of psychotherapy is heavily reliant on the quality of the therapeutic alliance. Theorists such as Bordin conceptualized this collaboration between patient and therapist as consisting of 3 core elements: a mutual agreement on the treatment goals, a shared understanding of the tasks involved, and the development of a bond built on trust and respect (Bordin 1979, 1994). This view is supported by Andrade-González, who argues that the strength of this alliance is a direct indicator of positive therapeutic outcomes, reinforcing its critical role in facilitating meaningful change (Andrade 2024). Regarding treatment duration, there is no universally set number of sessions due to the variety of therapeutic approaches employed. Recent research, such as that by Steffen and Anderson (Steffen and Anderson 2025), emphasizes that the therapy’s effectiveness depends not just on its duration but significantly on the strength of the therapeutic relationship and how we emotionally process our experiences. Their work highlights that our initial interpretation of events is predominantly emotional rather than rational. This view is perhaps one of the main reasons that could explain the results obtained after 3 sessions.

Additionally, the Program has developed tools, manuals, and publications available to all professionals and has created a Postgraduate course and Master’s degree in PSS care – both unique in our context – which have collectively trained over 200 professionals.

Based on the overall results, including effectiveness and resources produced, we consider that the Program has achieved its main aims of designing, developing, and generating experience and evidence about a care model to respond to the PSS needs of patients with advanced chronic conditions. In this regard, LCF, which considered the program catalytic, will assume the Program until the administration is able to fund the psychosocial care. This model might be adopted elsewhere and extended as a right and universal coverage.

### Strengths and limitations of the program and this report

The output and outcomes data associated with the Program are collected in a unique shared online information system, allowing a systematic evaluation using a quantitative and qualitative design, which generates experience and real-world evidence (National Institute for Health and Care Excellence 2022). This unique information system and the comprehensive evaluation encompassing multiple methodologies are among the program’s major strengths. The methods used are robust; however, a potential bias due to the quasi-experimental methodology used for the teams’ self-assessments cannot be ruled out. Moreover, during the initial phases of implementation of the Program, the evaluation was based on non-validated tools. Nevertheless, as shown in this study, numerous validated instruments have been implemented in subsequent phases.

In conclusion, a care Program based on dedicated support teams represents a feasible and effective model for organizing the PSS dimension of care for PACC and their relatives. The collaborative framework between public health services and non-profit foundations is effective, efficient, and feasible. The Program’s main challenge entails scaling this model of care and organization within the system to achieve universal coverage and integrate the PSS elements of care as an essential component of any palliative care model through advocacy to policymakers and society, and by disseminating results.

## Data Availability

The datasets generated and/or analyzed during the current study are available from the corresponding author on reasonable request.
