# Comprehensive psychosocial and spiritual care of people with advanced chronic conditions: The experience of La Caixa foundation program at 15 years – CORRIGENDUM

**DOI:** 10.1017/S1478951526101989

**Published:** 2026-03-31

**Authors:** Xavier Gomez-Batiste, Rosa Montoliu, Montserrat Codinachs, Cristian Tebé, Jorge Maté-Mendez

**Keywords:** Psychosocial and spiritual care, advanced chronic patients, care model, psychosocial interventions, real-world, corrigendum

The Author apologises for the following errors:

1. For omitting **Maria Joao Pinheiro**, and misspelling **Blanca Zorita** in the Acknowledgements section of the published article. The Acknowledgements should read as follows:

**Acknowledgments.** The authors thank the contribution of the team of “La Caixa” Foundation Program for the Comprehensive Care of People with Advanced Chronic Conditions, including Iciar Ancizu, Rafael Castell, Mireia Guardiola, Mireia Ingelmo, Ana Caterina Guillot da Costa, Jordi Claret, Belen Monge, Paula Aparicio, Maria Joao Pinheiro and Blanca Zorita. The authors would like to thank the i2e3 Procomms team (Barcelona, Spain) and especially, Sara Cervantes, Ph.D., for providing medical writing support during the manuscript preparation.

1. For misspelling the surname of co-author Jorge Maté-Mendez. It should be: Jorge Maté-Méndez
